# Swine influenza A virus: challenges and novel vaccine strategies

**DOI:** 10.3389/fcimb.2024.1336013

**Published:** 2024-04-03

**Authors:** Erika Petro-Turnquist, Matthew J. Pekarek, Eric A. Weaver

**Affiliations:** ^1^ Nebraska Center for Virology, University of Nebraska-Lincoln, Lincoln, NE, United States; ^2^ School of Biological Sciences, University of Nebraska-Lincoln, Lincoln, NE, United States

**Keywords:** swine influenza A virus, inactivated virus, replicon particle, live attenuated virus, DNA, vectored, computationally designed vaccines, nanovaccine

## Abstract

Swine Influenza A Virus (IAV-S) imposes a significant impact on the pork industry and has been deemed a significant threat to global public health due to its zoonotic potential. The most effective method of preventing IAV-S is vaccination. While there are tremendous efforts to control and prevent IAV-S in vulnerable swine populations, there are considerable challenges in developing a broadly protective vaccine against IAV-S. These challenges include the consistent diversification of IAV-S, increasing the strength and breadth of adaptive immune responses elicited by vaccination, interfering maternal antibody responses, and the induction of vaccine-associated enhanced respiratory disease after vaccination. Current vaccination strategies are often not updated frequently enough to address the continuously evolving nature of IAV-S, fail to induce broadly cross-reactive responses, are susceptible to interference, may enhance respiratory disease, and can be expensive to produce. Here, we review the challenges and current status of universal IAV-S vaccine research. We also detail the current standard of licensed vaccines and their limitations in the field. Finally, we review recently described novel vaccines and vaccine platforms that may improve upon current methods of IAV-S control.

## 1 Introduction

Influenza A virus (IAV) is a common pathogen caused by *Alphainfluenza virus* within the family of *Orthomyxoviridae.* IAV is a single-stranded negative-sense RNA virus with 8 genome segments. Each segment encodes one or more viral proteins, including the hemagglutinin (HA), neuraminidase (NA), nonstructural 1 (NS1), nonstructural 2 (NS2), nucleoprotein (NP), matrix 1 (M1), polymerase acidic (PA), polymerase basic 1 (PB1) and polymerase basic 2 (PB2) proteins (Howley, 2021). The error-prone RNA polymerase leads to high rates of mutations, known as genetic drift (Heaton et al., 2013). During co-infections with multiple strains of IAV, the segmentation of the genome allows for gene reassortment, referred to as genetic shift (Vincent et al., 2014). IAVs are classified into different subtypes based on the antigenic differences of the surface HA and NA proteins. Currently 18 different HA subtypes and 11 different NA subtypes have been identified. IAV infects a variety of species, which ranges from ducks, geese, and waterfowl to humans, equine, dogs, and pigs (Howley, 2021). In swine populations, the subtypes H1N1, H1N2, and H3N2 are endemic worldwide. Clinical symptoms of swine influenza A virus (IAV-S) typically manifest as pyrexia, anorexia, lethargy, coughing, labored breathing, and respiratory distress (Detmer, 2016). Though IAV-S can cause high levels of morbidity in infected herds, this usually does not translate to high rates of mortality. However, co-infections of IAV-S with other pathogens within the Porcine Respiratory Disease Complex (PRDC)- such as *Mycoplasma hyopneumoniae* (Thacker et al., 2001), porcine reproductive and respiratory syndrome virus (PRRSV) (Thacker, 2001), and porcine circovirus 2 (PCV2) (Ellis et al., 2004)- can lead to high rates of mortality. This pathogenesis risk can impose a significant economic burden on the pork industry, with current estimates indicating $1-$5 lost per pig each year (Moraes et al., 2023). Considering global pork production produces ~700 million hogs per year, IAV-S infection results in a significant impact on the global economy every year.

IAV-S is also considered a zoonotic pathogen because it can efficiently transmit between swine and humans. A recent meeting of the One Health initiative ranked zoonotic IAV as the top priority disease due to its pandemic potential. Swine were also recognized as a major host reservoir for the evolution of novel, pandemic IAV at the same workshop (One Health, Zoonotic Disease Prioritization for Multi-Sectoral Engagement in the United States (cdc.gov)). Swine play a unique role in the evolution of pandemic IAV due to their susceptibility to swine, avian, and human IAV. While avian species have a high prevalence of α-2,3-linked sialic acid receptors in their gastrointestinal tract and humans have α-2,6-linked sialic acid receptors in their respiratory tract, swine respiratory tracts contain both types of sialic acid linked receptors (Nelli et al., 2010). Co-infection, or “super infection”, with multiple strains of IAV from different species hosts can facilitate the swapping of gene segments and the development of new gene constellations that can then be transmitted to immunologically naive humans (Ito et al., 1998). The 2009 H1N1 “swine flu” pandemic represents an example of gene reassortment in pigs and zoonotic transmission to susceptible human populations (Smith et al., 2009). This novel IAV was initially recognized in Mesoamerica, then quickly spread and infected ~24% of the global population within the first year after its emergence (Van Kerkhove et al., 2013). This zoonotic transmission event established a new and stable lineage of H1 in humans, known as H1N1pdm09, that completely replaced the previously circulating H1N1 lineage.

Due to the significant financial burden and zoonotic potential of IAV-S, development of improved prevention strategies against influenza in swine is critical. Current prevention strategies used to control IAV-S include stringent biosecurity measures in the pork industry, appropriate flow of pigs during weaning and grow-stages of production, identification of circulating strains through surveillance, and vaccination. However, IAV-S prevention is still hampered with challenges. A recent survey of U.S. veterinarians reports that 89.4% of veterinarians consider IAV-S as one the top three health challenges facing the swine industry and 67% of surveyed veterinarians see IAV-S as an increasing threat in pig populations (Moraes et al., 2023). Despite recent improvement of non-invasive prevention strategies, vaccination is still the primary method of IAV-S control. While commercially available vaccines are used in over 80% of U.S. breeding herds, 74.2% of veterinarians at these sites believe the swine industry needs new or novel vaccine platforms (Moraes et al., 2023). Clearly, there is a dire need for improved vaccines against IAV-S. However, there are extensive challenges in developing broadly cross-protective vaccines against IAV-S. Here, we review the current challenges in developing a universal IAV-S vaccine. Further, we discuss the limitations of current licensed vaccines in reducing IAV-S burden. Finally, we detail novel experimental vaccines and vaccine platforms that may lead to advancements in protection against IAV-S.

## 2 Current challenges in developing a universal IAV-S vaccine

### 2.1 Genetic diversity of IAV-S

Several decades of unharnessed transmission between humans, birds, and pigs has fostered tremendous diversification of IAV-S in swine populations. While only three subtypes consistently circulate in swine, there is substantial genetic and antigenic diversity within and between each subtype (Walia et al., 2019; Anderson et al., 2021). The continued antigenic drift and shift has resulted in the evolution of multiple genetically diverse H1 and H3 lineages that have remained endemic in swine. Enhanced efforts into worldwide IAV-S surveillance have been an invaluable tool in understanding the kinetics of reassortment and circulation of IAV-S in different regions and at different times. The genetic evolution and diversity of IAV-S poses a significant challenge in developing a universal IAV-S vaccine and have been reviewed in detail elsewhere (Ma, 2020; Anderson et al., 2021). Here, we briefly overview the trends of genetic diversity in H1 and H3 IAV-S and the impacts on universal IAV-S vaccine development.

#### 2.1.1 H1 IAV-S

**Figure 1:**
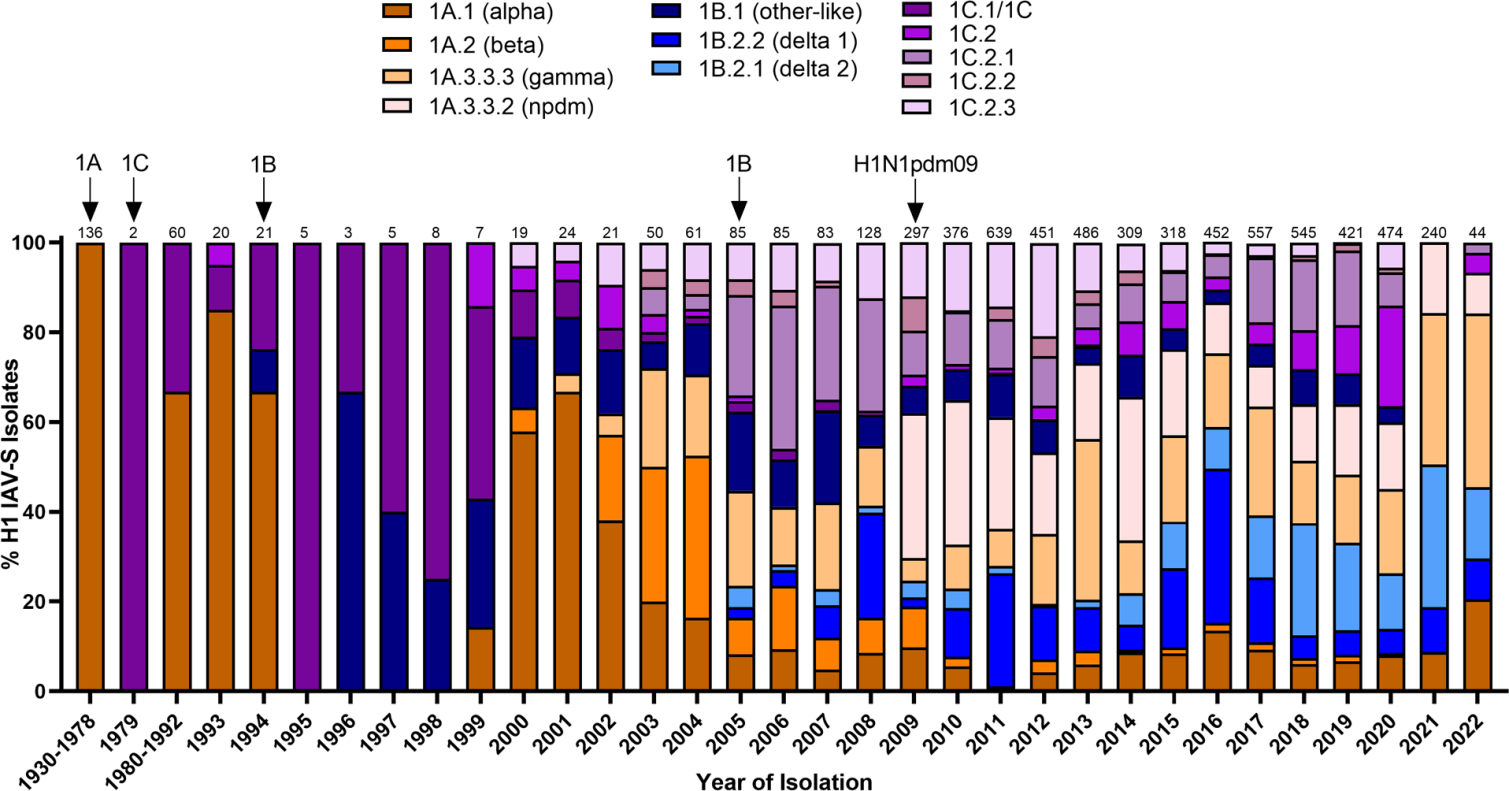
Temporal patterns of H1 IAV-S in worldwide swine populations from 1930-2022. All unique swine H1 IAV-S strains were downloaded from the Bacterial and Viral Bioinformatics Resource Center (Bacterial and Viral Bioinformatics Resource Center | BV-BRC (accessed on 14 October 2023)) and classified into H1 U.S. and global phylogenetic clades. Temporal patterns of diversity are visualized by year and the proportion of each designated clade was calculated based on total number of accessions submitted for a given year. Total number of accessions submitted per year/timeframe are indicated above each bar. Cross-species transmission events (human-to-swine or avian-to-swine) are indicated with arrows and the respective clade deposition.

Swine influenza A viruses with an H1 HA protein are classified into 3 different lineages based on regional occurrence and genetic similarity. While clinical symptoms consistent with influenza infections were observed in pigs as early as 1918, IAV-S was not isolated from swine populations until 1930 (Shope, 1931). There are currently three recognized H1 IAV-S lineages; 1A, 1B, and 1C. Reverse-zoonotic transmission of the H1N1 1918 Spanish Flu led to the establishment of the classical swine lineage (1A) in pigs. Repeated deposition of human-seasonal IAV in the 1990s and early 2000s established the human-seasonal (1B) lineage (Brown et al., 1995; Vincent et al., 2009; Lorusso et al., 2011; Nelson et al., 2015). Spillover of IAV from wild birds in the 1970s resulted in the Eurasian avian (1C) lineage in pigs (Guan et al., 1996). Finally, spillback of the human H1N1pdm09 in the years following the worldwide pandemic resulted in an established of new pandemic (npdm) clade in swine populations (**Figure 1**) with evidence of frequent human-to swine spillover events consistently occurring since 2009 (Markin et al., 2023). The 1A and 1B lineages are further classified into α (1A.1), β (1A.2), γ (1A.3), npdm (1A.3.3.2), δ1 (1B.2.2), and δ2 (1B.2.1) clades, with additional sub-clade classification based on region of origin (Anderson et al., 2016). Analysis of the average pairwise nucleotide distances within the 1A, 1B, and 1C lineages indicate that the genetic diversity of the HA protein can extend up to 14.4%, 16.1%, and 10.3% within each respective lineage (Anderson et al., 2016) and 35.9% between lineages (Anderson et al., 2013). Antigenic mapping of H1 has further revealed that one or two amino acid mutations in the HA1 domain can significantly impact the ability for antibodies to recognize and neutralize viruses in divergent clades (Lorusso et al., 2011). This provides an additional barrier to developing a universal vaccine against IAV-S. Indeed, despite significant efforts in optimizing vaccine composition to elicit broad protection against antigenically diverse H1 IAV-S, both H1N1 and H1N2 subtypes have remained the most predominantly circulating subtypes of IAV-S in U.S. swine populations for over a decade (Walia et al., 2019). Further advancements in vaccine efficacy to address the H1 IAV-S will be needed to better control its circulation among U.S. herds.

#### 2.1.2 H3 IAV-S

**Figure 2:**
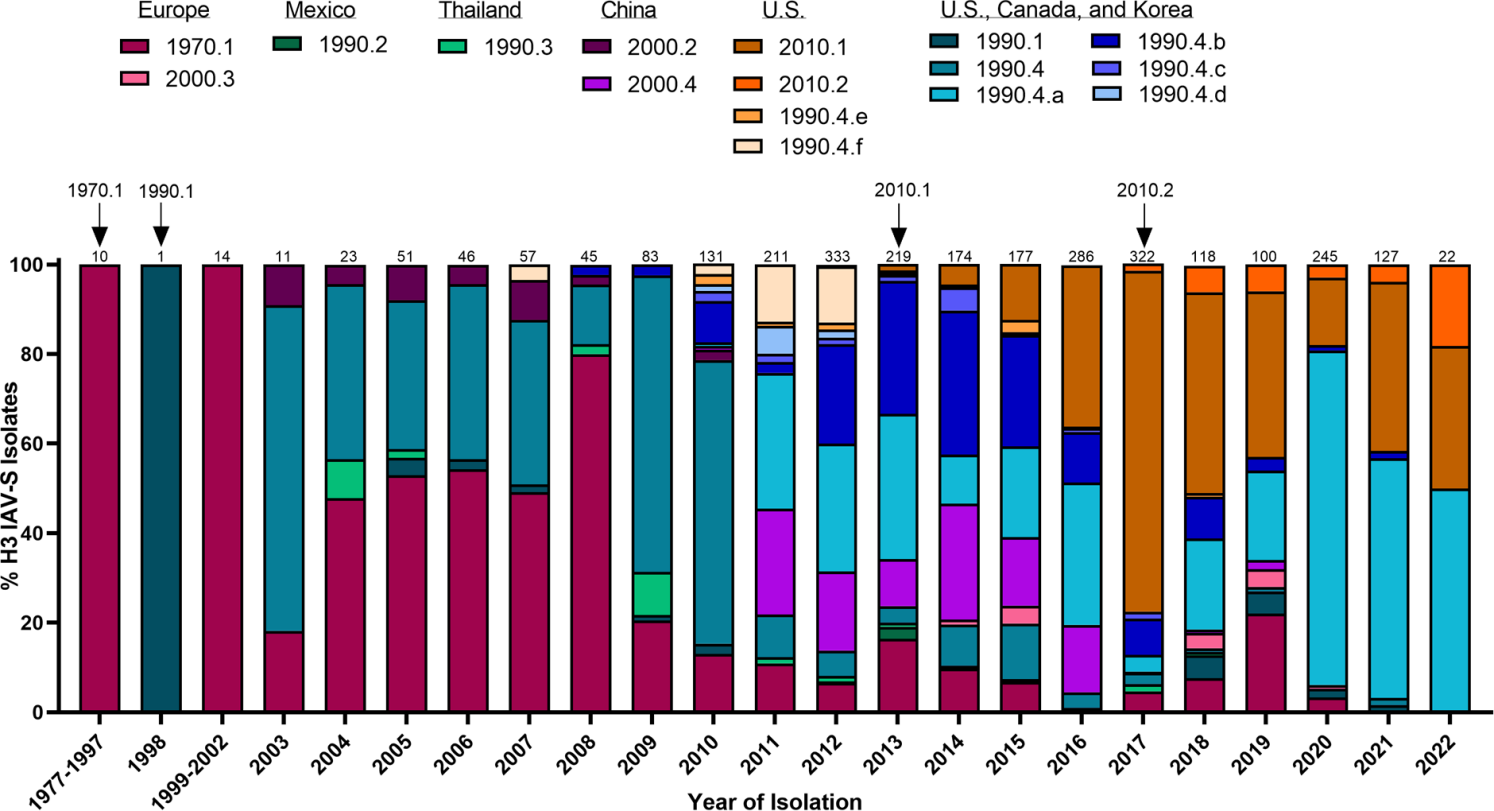
Temporal patterns of H3 IAV-S in worldwide swine populations from 1977-2022. All unique swine H3 IAV-S strains were downloaded from the Bacterial and Viral Bioinformatics Resource Center (Bacterial and Viral Bioinformatics Resource Center | BV-BRC (accessed on 16 October 2023)) and classified into H3 U.S. and global phylogenetic clades. Temporal patterns of diversity are visualized by year and the proportion of each designated clade was calculated based on total number of accessions submitted for a given year. Total number of accessions submitted per year/timeframe are indicated above each bar. Cross-species transmission events (human-to-swine) are indicated with arrows and the respective clade deposition.

H1N1 stably circulated in pigs for several decades until the detection of a novel H3N2 (1970.1) in European pig populations in the 1970s. The 1970.1 clade IAV-S subsequently reassorted with 1C-lineage H1N1 in the 1980s. In 1998, a novel triple-reassortant H3N2 was identified in the U.S. (**Figure 2**). The triple-reassortant internal gene (TRIG) constellation contained the NP, M, and NS gene segments derived from the circulating classical swine lineage, the PB1, HA, and NA genes from human seasonal IAV, and the PB2 and PA gene segments from avian IAV (Zhou et al., 1999). H3N2 has since been established in pig populations and is categorized into phylogenetic clades; Cluster I (1990.1), Cluster II (1990.2), Cluster III (1990.3), and Cluster IV (1990.4) (Walia et al., 2019) with persistence of the TRIG constellation detected in both H3N2 and H1N1 IAV-S (Vincent et al., 2008). Continued genetic diversification of cluster IV (1990.4) IAV-S has resulted in additional sub-cluster IAV-S (1990.4.a-1990.4.f) that have been detected in the U.S. Canada, and Korea. In the 2010 decade, spillover of IAV from humans to swine established novel H3 IAV-S in Vietnam (Ngo et al., 2012), China (He et al., 2018), and established new lineages known as the 2010.1 and 2010.2 human-like lineages (Zeller et al., 2018) (**Figure 2**). Notably, while H1 HA tends to demonstrate more promiscuity during reassortment events in swine, H3 HAs almost exclusively pair with N2 NA lineages of human seasonal virus origin (Rajao et al., 2015). The mechanisms governing this preferential reassortment are currently unknown. While H3 does not demonstrate as expansive genetic diversity as H1 IAV-S, there is still up to 12.4% sequence diversity between H3 Clusters I-IV (Anderson et al., 2013). The lack of control over H3 IAV-S, despite lower overall diversity than H1 IAV-S, indicates that a different optimal strategy is needed to achieve the goal of creating a universal IAV-S vaccine.

### 2.2 Maternally derived antibodies

In large-scale pork production, gilts and breeding sows are often vaccinated prior to farrowing to optimize the transfer of maternally derived antibodies (MDA) to suckling piglets. These passively acquired immune complexes can be taken up from the colostrum into the bloodstream within the first 24 hours after birth. Circulating MDA can protect neonatal piglets against important infectious diseases prior to complete immune maturation (Bourne and Curtis, 1973). While homologous MDA can protect piglets from IAV-S-associated clinical disease (Loeffen et al., 2003; Choi et al., 2004; Kitikoon et al., 2006; Sandbulte et al., 2014) and decrease transmission of antigenically similar IAV-S (Corzo et al., 2014; Cador et al., 2016; Chamba Pardo et al., 2019), heterologous MDA do not provide protection against antigenically distinct IAV-S (Allerson et al., 2013; Rajao et al., 2016). Further, MDA have even been described to exacerbate disease after heterologous infection in weaned piglets (Pyo et al., 2015; Rajao et al., 2016), providing an additional barrier to achieving protection against IAV-S. Importantly, both whole inactivated virus (WIV) and alphavirus replicon particle (RP) IAV-S vaccine platforms are often inhibited by MDA (Bosworth et al., 2010; Sandbulte et al., 2014). While MDA against IAV-S gradually wane over time, studies show that these can last in nursery pigs for up to 14 weeks after birth (Markowska-Daniel et al., 2011). As a result, herds with a high prevalence of endemic IAV-S often delay vaccination until 12-16 weeks of age to limit MDA interference to immunization. This lag in vaccine administration can often result in significant circulation of IAV-S among susceptible pigs with suboptimal humoral immunity against IAV-S. Consequently, recent efforts in creating a universal IAV-S have shifted focus towards creating a vaccine that is not susceptible to MDA interference and can induce broadly cross-protective antibody responses to limit exacerbation of disease after passive transfer to suckling piglets. Currently there are no licensed vaccines that are resistant to MDA interference, and experimental vaccines must consider these passive immune complexes during development and clinical testing.

### 2.3 Vaccine-associated enhance respiratory disease

Vaccine-associated enhanced respiratory disease (VAERD) is a phenomenon initially described after inactivated RSV vaccination in young children (Kim et al., 1969; Delgado et al., 2009) and has recently been observed in pigs after immunization with inactivated IAV-S. VAERD is hallmarked by high concentrations of cross-reactive, but non-neutralizing antibodies directed towards an immunodominant epitope in the HA2 stalk domain of the HA protein. VAERD was initially described when pigs were immunized with an inactivated virus targeting a δ1-H1N1 clade virus and subsequently challenged with an H1N1pdm09 IAV-S (Gauger et al., 2011). In the absence of neutralizing antibodies against the HA1 head domain, stalk-directed antibodies were shown to facilitate enhanced viral infection of Madin-Darby canine kidney (MDCK) cells *in vitro* (Khurana et al., 2013) and rapidly induce dysregulated levels of proinflammatory cytokines in the lungs of infected pigs (Gauger et al., 2011, 2012). Further recruitment of inflammatory cell populations, such as neutrophils and macrophages, results in collateral damage in the lungs and enhanced respiratory disease (Gauger et al., 2012). The precise mechanisms of how the HA2 stalk-directed antibodies facilitate enhanced membrane fusion are currently unknown, but it is hypothesized that bound HA2 antibodies may be enhancing the rate of membrane fusion in endosomal compartments during the initial stages of infection. However, additional research into these mechanisms is necessary to confirm this hypothesis. Given that VAERD is mediated by stalk-directed antibodies, chimeric HA strategies currently in development against human IAV (Bullard and Weaver, 2021) likely would not be beneficial in the development of a universal IAV-S vaccine. Further, this phenomenon demonstrates that cross-reactive antibodies should be thoroughly examined for the potential to elicit VAERD upon heterologous challenge and exemplifies an additional challenge in developing a universal IAV-S vaccine.

### 2.4 Vaccine-induced humoral and cell-mediated immunity

#### 2.4.1 Humoral immunity

Neutralizing antibody responses are a well-established correlate of protection against IAV-S. Neutralizing antibodies can provide sterilizing immunity by targeting the immunodominant surface HA protein to prevent entry into host cells (Johansson et al., 1987). Consequently, current vaccines primarily aim to elicit neutralizing antibody responses directed towards the HA protein. While the precise kinetics of antibody responses after vaccination in pigs are relatively understudied, experimental infection with IAV-S shows that HA-specific IgM and IgG antibodies in serum peak by 7 and 25 days after infection, respectively (Edmans et al., 2020). In contrast, local IgA and IgG can be detected as early as 4-5 days after IAV-S infection (Lee et al., 1995; Larsen et al., 2000). Recent research correlated local and systemic antibodies elicited after different routes of immunization in pigs and observed that either local or systemic neutralizing antibody responses can provide protection against IAV-S infection (Vatzia et al., 2023). Cross-protective antibody responses against genetically divergent HA proteins are dictated by the overall genetic similarly of the HA protein and how well the targeted epitopes match (Reeth et al., 2004; Kreijtz et al., 2011). Non-neutralizing IgG subclasses can participate in various Fc-mediated effector functions such as complement-dependent cellular cytotoxicity (CDCC), antibody-dependent cellular cytotoxicity (ADCC), and antibody-dependent cellular phagocytosis (ADCP) (Paudyal et al., 2022) and may contribute to clearance of IAV-S (Kreijtz et al., 2011). Assessment of NP, M, and NA targeting antibody responses also suggests that these may play a role in providing partial protection against heterologous IAV-S infection (Heinen et al., 2000, 2001; Kitikoon et al., 2008; Sandbulte et al., 2016) but these immunogens often must be delivered in combination with an HA to achieve complete protection (Vander Veen et al., 2013; Borggren et al., 2016; Wymore Brand et al., 2022). Current vaccine studies typically only analyze the neutralizing capacity of antibodies elicited after vaccination, however further research into the targeted immunodominant B cell epitopes in the HA, NA, NP, and M IAV-S proteins may help to uncover potentially broadly neutralizing antibodies and improve efforts of developing a vaccine with universal protection.

#### 2.4.2 Cell-mediated immunity

Neutralizing antibody responses targeting the HA protein have historically been a focal point of most vaccine studies. However, newer developments are appreciating the importance of cell-mediated immune responses against IAV-S. In the absence of neutralizing antibodies, cytotoxic T cells are recruited to the site of infection to recognize IAV-S infected cells and produce cell-lysing perforin and granzyme molecules to efficiently eliminate infected cells (Sun and Braciale, 2013; Tchilian and Holzer, 2017) through the activation of apoptosis (Zychlinsky et al., 1991; Keefe et al., 2005). Proliferating Ki67^+^ CD4^+^ T cells can be detected in the tracheobronchial lymph nodes (TBLN) of experimentally infected pigs as early as 4 days after infection (Talker et al., 2016), and IFN-γ secreting CD8^+^ T cells can migrate to the lungs and bronchioalveolar lavage (BAL) by 6 days after infection (Khatri et al., 2010; Talker et al., 2016). Protection against antigenically distinct IAV-S after infection is often mediated by cross-reactive T cell responses to conserved internal proteins and conserved regions of the surface viral glycoproteins (Van Reeth et al., 2009; De Vleeschauwer et al., 2011; Hillaire et al., 2011). However, the induction of cell-mediated immune responses in vaccinated pigs is an understudied field of research. Further research into this area and the induction of site-specific T cell responses will improve vaccination methods against IAV-S in pigs. Further, identifying potentially immunodominant T cell epitopes may contribute to the development of a broadly protective IAV-S vaccine.

## 3 Licensed vaccines against IAV-S

### 3.1 Whole inactivated virus vaccines

Whole inactivated virus (WIV) vaccines are currently the most commonly used platform to vaccinate pig populations against IAV-S (Sandbulte et al., 2015). WIV vaccines are delivered intramuscularly, often target multiple IAV-S strains, and include an oil-in-water adjuvant to enhance the immune response to the delivered antigens. Commercial WIV vaccine production typically relies on regional and national surveillance data to identify circulating IAV-S clades and guide strain selection (Sandbulte et al., 2015). Once wildtype field isolates representing the genetic and antigenic diversity of IAV-S in swine populations are identified, the chosen strains are grown in embryonated chicken eggs, inactivated by formaldehyde or β-propiolactone treatment, and purified for final vaccine formulation (Herrera-Rodriguez et al., 2019). However, due to long production times, WIV vaccines are infrequently updated and commonly fail to provide broad protection. WIV vaccines are most well-known for inducing antibody responses, but typically do not induce strong cell-mediated immune responses because they are a form of non-replicating vaccine. Indeed, this vaccine modality can induce strong antibody responses and protect against homologous strain challenge, but limited protection is typically observed after challenge with a heterologous strain (Abente et al., 2018). This strain-specific immune induction is due to the extensive genetic diversity of IAV-S and low cross-reactive T cell responses, providing an additional challenge in creating a universal WIV IAV-S vaccine. To improve the protective efficacy of commercial WIV vaccines, pork producers are opting to utilize autogenous, or “custom-made”, vaccine formulations. In the U.S. ~50% of pork producers utilize autogenous vaccine formulations to improve herd protection (Moraes et al., 2023). Autogenous vaccines can be useful when commercial WIV vaccines are not antigenically matched to circulating strains in a given herd. However, autogenous vaccines are hampered by a significant lag time during IAV-S detection, isolation, development, and administration. Further, WIV vaccines are well-known to be inhibited by MDA and can have dramatically reduced efficacy if administered before MDA. Due to the challenges associated with WIV vaccines, newer vaccine strategies against IAV-S have been developed and are becoming increasingly used in worldwide swine populations.

### 3.2 Alphavirus Replicon Particle (RP) Vaccines

Alphavirus replicon particle (RP) vaccines represent one of the most advanced vaccine technologies against IAV-S. Replication-defective RP vaccines are developed from an attenuated Venezuelan equine encephalitis virus (VEEV) (Erdman et al., 2010). VEEV has an ~11kb positive-sense RNA genome with two open reading frames. The 5’ open reading frame encodes the four nonstructural proteins (nsp1-4) while the 3’ open reading frame encodes the four viral structural proteins necessary for capsid assembly and packaging. Upon infection, the VEEV non-structural genes are translated from the positive-sense genome to develop genomic RNA and 26S subgenomic RNA. The 26S subgenomic RNA subsequently serves as a template to translate the viral structural genes (Vander Veen et al., 2012a). When the viral structural genes are supplied *in trans*, these regions can be replaced with one or more desired immunogens to develop a replication-defective RP vaccine (Pushko et al., 1997; Kamrud et al., 2010). RP vaccines have previously been shown to be well tolerated and induce robust hemagglutination inhibition (HI) antibody responses against H1N1pdm09 (Vander Veen et al., 2009) and H3N2 (Vander Veen et al., 2012b) IAV-S to reduce lung lesions and viral shedding after challenge. Importantly, vaccination with an H3N2 NP-expressing RP has also been shown to protect against viral shedding and viral load in the lungs after heterosubtypic H1N1pdm09 challenge (Vander Veen et al., 2013) and do not induce VAERD after heterologous challenge (Wymore Brand et al., 2022). Due to this success, RP vaccines were approved by the USDA for use in swine against IAV-S in U.S. swine populations. RP vaccines have been further approved as autogenous vaccines that encode HA proteins representing the genetic diversity of circulating IAV-S within a given herd. However, there are still inherent challenges with RP vaccines. First, though RP vaccines can be matched to circulating strains identified in a herd, they are susceptible to inhibition by MDA (Bosworth et al., 2010). Second, similar to autogenous WIV vaccines, autogenous RP vaccines have a significant lag time in development and may be costly to produce. Nonetheless, since the licensing of the RP vaccine platform in 2012, this vaccine platform has gained popularity and may be the best option for IAV-S control in swine populations.

### 3.3 Live attenuated influenza virus vaccines

Live attenuated influenza virus (LAIV) vaccines are at the forefront of novel vaccine development against IAV-S. In humans, LAIV vaccines were initially licensed in the early 2000s and are currently under intense development to protect pigs against IAV-S. The attenuation of LAIV vaccines aims to restrict replication after immunization and decrease the likelihood of genetic reassortment between the LAIV vaccine and circulating field strains. LAIV vaccines are typically administered intranasally to facilitate limited replication in the upper respiratory tract, induce site-specific mucosal antibody responses, and activate systemic cell-mediated T cell responses. Initial LAIV development has focused on truncation of the NS1 viral protein. NS1 modulates host type I interferon responses, and deletion of the first 126 amino acids (NS1Δ126) of this protein prevents its antagonistic function, resulting in a highly attenuated virus mutant (Solorzano et al., 2005). The strain chosen for development was the prototype H3 IAV-S; A/swine/Texas/4199-2/1998 (TX98NS1Δ126). During initial experimental testing, intratracheal vaccination with the TX98NS1Δ126 LAIV vaccine resulted in complete protection against homologous challenge and partial protection against heterosubtypic challenge. This partial protection against heterologous was likely mediated by high induction of mucosal IgA responses (Richt et al., 2006) and cell-mediated immune (CMI) responses to internal proteins, though the induction of CMI responses was not analyzed until later. Later assessment of NS1Δ126 intranasal immunization compared to intramuscular immunization demonstrated that two doses of intranasal immunization completely protected against homologous viral challenge and nearly complete protection against heterologous challenge (Vincent et al., 2007). Protection against heterologous challenge was later shown to be mediated by high mucosal IgA and IgG responses and priming of CD4^+^CD8^-^, CD4^+^CD8^+^, and CD4^-^CD8^+^ IFN-γ producing T cells after immunization (Kappes et al., 2012). Importantly, a comparative study analyzing 1 dose of intranasal LAIV vaccination compared to 2 intramuscular doses of TX98 WIV vaccination in the presence of MDA demonstrated that LAIV provided complete protection against homologous TX98 challenge and reduced lung damage and viral loads by 5 days after infection. While the levels of serum IgG and IgA responses were reduced in the presence of MDA, these data indicate that LAIV vaccination can induce local immune responses that remain robust in the presence of MDA (Vincent et al., 2012) and is not susceptible to interference of MDAs in piglets (Genzow et al., 2018).

These promising experimental results eventually led to the licensure of the NS1Δ126 LAIV in 2017 and the widespread use in U.S. swine populations. The marketed LAIV contained two subtypes of IAV-S; H1N1 strain A/swine/Minnesota/37866/1999 and the H3N2 strain A/swine/Texas/4199-2/1998 and was licensed for piglets as young as 2 days of age. While experimental transmission studies showed limited LAIV shedding after vaccination, minimal transmission to non-vaccinated pigs, and no evidence of reassortment between endemic IAV-S (Lopez Moreno et al., 2021), later surveillance of U.S. swine populations revealed evidence of reassortment between the LAIV vaccine virus and circulating endemic H1 and H3 field strains (Sharma et al., 2020), indicating that experimental conditions cannot always recapitulate field conditions. This licensed LAIV was subsequently removed from commercial use in 2020 and highlights the need for continued IAV-S surveillance to identify asymptomatically infected pigs and limit the risk of reassortment during vaccination.

## 4 Novel experimental vaccines against IAV-S

While vaccination plays a crucial role in preventing the spread of IAV-S in pigs, current vaccines are hampered by low efficacy and inadequate cross-protection, inhibition by MDA, slow production times, and questionable safety profiles. Consequently, researchers are developing novel and rationally designed vaccines to improve vaccination strategies in pigs. Ideally, a universal IAV-S vaccine should elicit a broad immune response that provides protection against antigenically divergent H1 and H3 IAV-S, will overcome MDA interference, has an optimal safety profile, and will not induce VAERD. Here, we review recently described experimental vaccines that are currently under investigation for the development of a universal IAV-S vaccine.

### 4.1 Experimental LAIV

#### 4.1.1 Elastase-dependent LAIV

**Figure 3:**
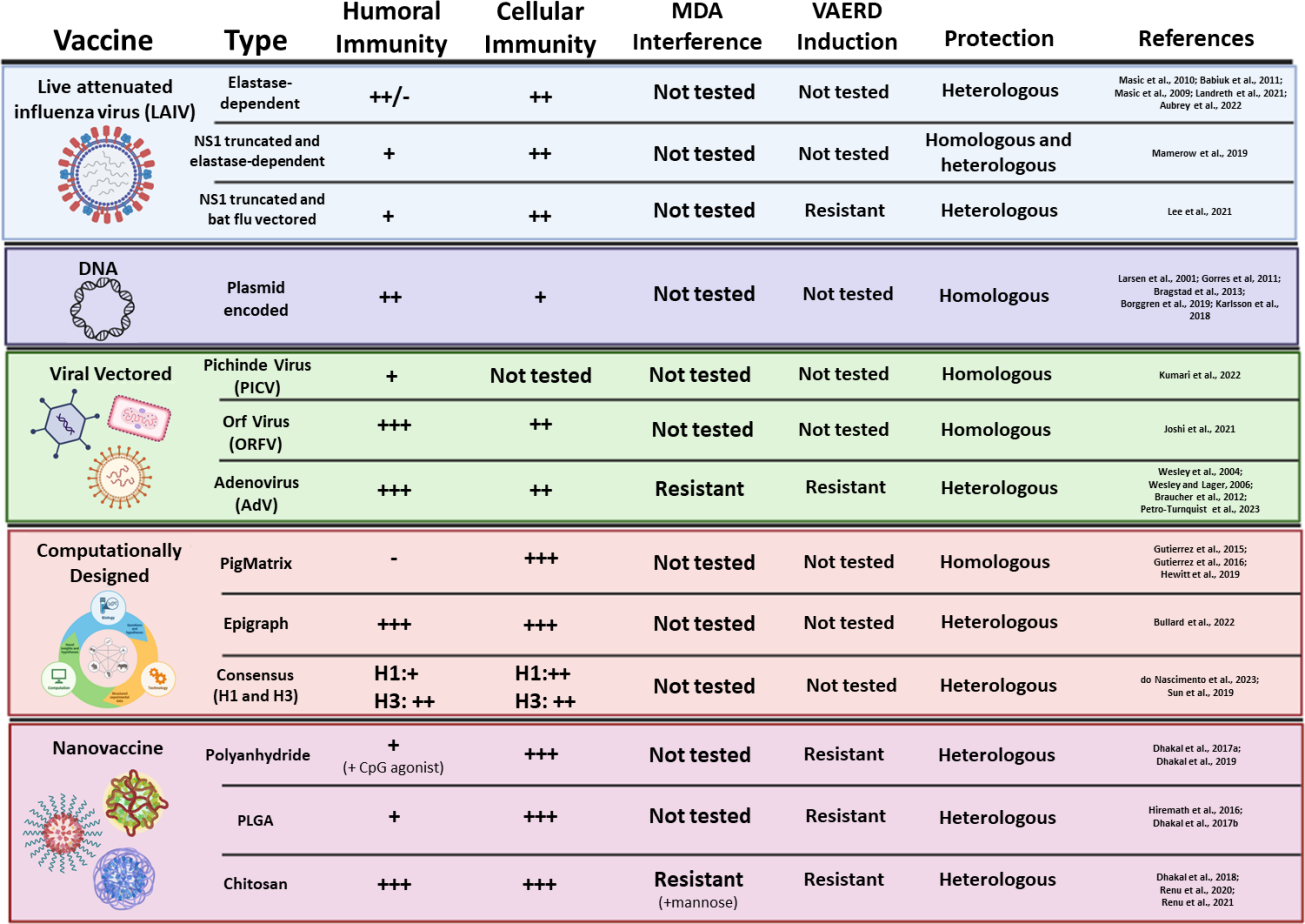
Novel experimental vaccine platforms against IAV-S. Vaccine type, platform, induction of humoral and cell-mediated immunity, inhibition by MDA, induction of VAERD, and protection against challenge are summarized for each novel vaccine platform.

To improve the safety profile of the recently redacted NS1 truncated LAIV, additional LAIV platforms are under intense investigation (**Figure 3**). Another method of influenza virus attenuation is mutating the surface HA protein to depend on elastase instead of trypsin for enzymatic cleavage during infection (Stech et al., 2005, 2011). Elastase is limited in the respiratory tract of pigs, which suggests that viral replication will be heavily restricted after vaccination and abrogate the possibility of reassortment with field strains. Indeed, Zhou and colleagues have previously shown the robust attenuation of this strategy *in vitro* and *in vivo* (Masic et al., 2009a, Masic et al., 2009b) and promisingly demonstrated that intranasal immunization with this LAIV platform provides protection against heterologous pre-pandemic H1N1 (Masic et al., 2010), pandemic H1N1 2009 (Babiuk et al., 2011), and H3N2 (Masic et al., 2009b). Recent analysis of a bivalent, elastase-dependent LAIV vaccine developed from H1N2 and H3N2 IAV-S field isolates demonstrated robustly neutralizing antibody responses against the homologous vaccine strains after two immunizations. However, analysis of neutralizing antibody responses against a heterologous H1N1 IAV-S isolate did not indicate robust cross-neutralizing antibody responses after the prime or boost immunizations. Further assessment of IFN-γ secreting T cells isolated from tracheobronchial lymph nodes after challenge showed that pigs immunized with the bivalent LAIV vaccine then challenged with a heterologous H1N2 virus resulted in significantly higher T cell responses than unvaccinated and unchallenged controls. While these results suggest that the bivalent LAIV vaccine may be able to induce T cells that are reactivated upon restimulation during challenge, this cannot be definitively concluded since IFN-γ T cell responses were not analyzed prior to challenge and remains an open question. Nonetheless, vaccination with the bivalent LAIV demonstrated protective efficacy through significant reduction of lung lesions and infectious virus titers in the lungs after challenge with homologous H1N2, homologous H3N2, and heterologous H1N2 IAV-S (Landreth et al., 2021). Subsequent analysis detailed protection of the bivalent, elastase-dependent LAIV against antigenically drifted H1N2 and H3N2 IAV-S. Though the drifted H1N2 and H3N2 isolates had mutations in several key H1 and H3 antigenic sites compared to the vaccine strains, the LAIV vaccine maintained protective responses against these clinical isolates through significant reduction of clinical disease, diminished lung lesions, and reduction of infectious virus in the lungs and nasal swabs (Aubrey et al., 2022). These data indicate that the bivalent, elastase-dependent LAIV has the potential to enhance cross-reactive responses against divergent IAV-S. Previous research against the NS1-truncated LAIV indicates that the bivalent elastase-dependent LAIV may not be susceptible to MDA, but further research is necessary to uncover this potential interference. Further, additional research detailing the potential for this platform to reassort with endemic IAV-S is necessary prior to full licensing of this novel vaccine platform.

#### 4.1.2 Double-attenuated LAIV

To piggyback off the previous success of the NS1 truncated LAIV, additional research has worked to enhance the safety profile by developing a double-attenuated LAIV vaccine against IAV-S (**Figure 3**). This strategy utilizes both truncation of the NS1 protein and an elastase-dependent HA mutant virus to produce the LAIV. Mamerow et al. recently characterized the immunogenicity and protective efficacy of this double-attenuated LAIV platform in pigs. Pigs immunized with the double-attenuated LAIV encoding an H1N1pdm09 IAV were completely protected against homologous (H1N1pdm09) and homosubtypic challenge (H1N1), and were partially protected against heterosubtypic challenge (H3N2). Interestingly, only moderate antibody responses were elicited after vaccination while CD4^+^ and CD8^+^ T cells were enriched after challenge (Mamerow et al., 2019). This suggests that protection elicited from this LAIV may be mediated through cell-mediated immune functions rather than antibody neutralization alone. While this platform is still in the beginning stages of development, further research assessing the multi-functional capacity of T cell elicited after vaccination may help to clarify the precise immunological mechanisms of protection elicited by this novel vaccine platform. Another method of double-attenuation is a recently described bat influenza virus vectored and NS1-truncated LAIV (**Figure 3**). Ma and colleagues have previously established that chimeric bat influenza viruses can be modulated to contain alternative HA or NA proteins (Juozapaitis et al., 2014; Zhou et al., 2014), but do not reassort with conventional IAV-S (Yang et al., 2017). This indicates that this LAIV could be an excellent platform to mitigate reassortment with field IAV-S while delivering immunogenic vaccine antigens. Recently, Lee et al. generated a LAIV using the HA and NA proteins of H3N2 TX98 and the 6 internal genes from an H17N10 bat IAV, where the NS1 protein was truncated. While the WIV TX98 induced higher serum IgG levels, the LAIV vaccine induced significantly higher levels of IFN-γ secreting T cells after heterologous challenge. Further, pigs vaccinated with the TX98 WIV demonstrated significant lung lesions by 3 days post challenge that were not present in the LAIV vaccinated pigs. Finally, LAIV immunization significantly reduced virus replication in the lungs and infectious virus from nasal secretions after heterologous challenge, while the WIV vaccine failed to reduce levels of infectious virus (Lee et al., 2021). Similar to other LAIV vaccines, this double-attenuated platform showed restricted replication after intranasal immunization, but still induced a robust IgG and IgA response in the lungs to enhance protective immunity at the site of infection. While these platforms are still in the experimental stages of development and require further assessment to determine field safety conditions, these preliminary results suggest that double-attenuated LAIV may be promising platforms that are robust against MDA, may have a significantly enhanced safety profile compared to previously described LAIVs, and can provide broad protection as a universal IAV-S vaccine.

### 4.2 DNA vaccines

Another vaccine platform currently under investigation against IAV-S are DNA vaccines. DNA vaccines are often delivered in the form of plasmids and encode one or more target genes. The host cells transfected by the plasmid transiently express the immunogen, which can lead to induction of both antibody- and cell-mediated immune responses (Dhama et al., 2008). This – combined with other properties of the DNA vaccine platform such as ease of manufacturing, no possible virulence reversions, immunogen carrying flexibility, and versatility of delivery mechanisms (Accensi et al., 2016; Ledesma-Feliciano et al., 2023) – makes DNA vaccines an attractive platform for livestock vaccine development. Indeed, DNA vaccines have been tested against a wide variety of swine viral pathogens, including PRRSV (Du et al., 2017; Cui et al., 2019; Zhao et al., 2022), PCV2 (Park et al., 2017; Hou et al., 2019), PEDV (Zhang et al., 2016), and CSFV (Markowska-Daniel et al., 2001; Ganges et al., 2005; Tarradas et al., 2010; Li et al., 2015). Swine influenza vaccines using a DNA platform have recently been studied to take advantage of the platform’s benefits. Typically, wild-type glycoprotein sequences are delivered as the vaccine antigens. Plasmid delivery of these antigens induces neutralizing antibodies at much higher titers after two immunizations with plasmid DNA (Macklin et al., 1998; Gorres et al., 2011; Bragstad et al., 2013; Borggren et al., 2016; Karlsson et al., 2018). When measured by HI assay, one study reported HI titers ≥ 100 against multiple strains after two DNA vaccine doses (Bragstad et al., 2013), which is well above the correlate of protection against human influenza infection (Hannoun et al., 2004). Interestingly, another study showed DNA prime vaccination followed by whole-inactivated virus boosting seemed to induce a greater HI response post-vaccination, but two doses of DNA vaccine led to greater levels of total neutralizing and HI antibody activity when vaccination was followed by challenge (Larsen et al., 2001). Despite the differences in vaccine composition, it is clear that multi-dose delivery of swine influenza DNA vaccine is sufficient to induce strong antibody responses in pigs.

In addition to inducing neutralizing antibodies, there is interest in inducing T-cell responses after vaccination to help identify and kill infected cells. Historically, this has not been a prominent focus of the field. However, Larsen, et al. reported low detection of virus-specific IFN-γ secreting cells after vaccination with 1 or 2 doses of plasmid DNA but observed an increase in IFN-γ secreting cells isolated from the spleen and nasal mucosa after live virus challenge (Larsen et al., 2001). Detection of IFN-γ secreting T-cells was also observed against the HAs encoded by multiple plasmids delivered together as a cocktail (Gorres et al., 2011). Interestingly, when internal genes were delivered alongside the HA and NA proteins, IFN-γ secreting cells targeting the NP and M1 genes were detected at a higher level after re-stimulation than HA-targeting IFN-γ secreting cells (Borggren et al., 2016). One limitation of the T-cell stimulation after vaccination across all 3 studies reporting this data is the absence of any heterologous T-cell response assessment. All the studies observed T-cell responses after vaccination and re-stimulation with the same immunogen that was delivered. Understanding the ability to induce cross-reactive T-cell responses would better assess the translatability into the field setting, where the circulating strains are not likely to be homologous to the vaccine immunogen.

In pigs a primary focal point of IAV-S DNA vaccine studies has been the assessment of protection from live influenza virus challenge. In studies that performed a challenge after immunization with wild-type influenza immunogens, the DNA vaccination led to lower viral titers observed over time after challenge (Larsen et al., 2001; Gorres et al., 2011; Bragstad et al., 2013; Karlsson et al., 2018). Most studies only compared the DNA vaccination to an unvaccinated control challenge to observe titers. However, Larsen, et al. found that pairing a prime DNA vaccination with a boost whole-inactivated vaccine dropped the infectious virus titer from the nasal secretions dramatically compared to two DNA vaccinations (Larsen et al., 2001). Investigation by Gorres, et al. into the effects of infection showed that the DNA vaccination prevented inflammatory cell infiltration and bronchiole cuffing observed by H&E staining when compared to the control group. Further, no detection of viral HA in the lung tissue as measured through immunohistochemistry after infection with A/CA/2009 or A/Ohio/2007 viral challenges (Gorres et al., 2011). Taken together, these studies suggest that DNA vaccination against swine influenza viruses can provide a strong barrier to infection in pigs. However, there is currently a lack of understanding of the protection afforded by DNA vaccination against heterologous challenge strains. Due to the importance of challenge strain relatedness to vaccine immunogen for inactivated swine influenza vaccines and its association with VAERD (Mancera Gracia et al., 2020), this remains a crucial unanswered question before DNA swine influenza vaccines are translated to larger scale use in the future.

Some additional studies have investigated how the benefits of the DNA vaccine platform can be used for swine influenza vaccines. Considerable efforts have gone into comparing traditional needle-syringe delivery with needle free delivery. When compared directly, no significant differences in antibody or T-cell responses could be detected between traditional delivery and needle-free vaccination (Gorres et al., 2011). Further, cross-reactive antibody responses could be detected against human influenza strains not included in the vaccination design after two doses of needle-free delivery of DNA vaccines (Borggren et al., 2016). Other studies have investigated alternative immunogen designs to improve upon the response to wild-type immunogen sequences. McCormick, et al. designed an immunogen sequence by “shuffling” the sequences of genetically distinct swine influenza strains to create a chimeric HA protein (Mccormick et al., 2015). After immunization in pigs, the authors reported HI titers ≥ 100 against not only the strains incorporated into the immunogen design, but also additional strains from other clades of swine H1 influenza (Mccormick et al., 2015). This study did not conduct a challenge experiment to determine protective efficacy from live influenza strains or assess T-cell responses after immunization of pigs. Another group has designed an alternative immunogen by generating a recombinant sequence based on conserved epitopes from H1, H5, and H7 influenza HA sequences or H1 HA alone (Sistere-Oro et al., 2019a, Sistere-Oro et al., 2019b). In one study, animals vaccinated with pre-existing MDAs to H3 HA had a significant increase in H3 specific HI antibodies after vaccination and increased the levels of total T-cells circulating after vaccination compared to unvaccinated pigs (Sistere-Oro et al., 2019a). The effect of vaccination on immune correlates was not as significant for naïve animals. However, the DNA vaccination led to trends of lower viral RNA detected in the lungs after challenge with either H1 or H3 IAV-S compared to unvaccinated animals regardless of previous influenza antibody circulation correlating with protection from challenge (Sistere-Oro et al., 2019a). A different study investigated whether vaccination with an H1 epitope conjugated with human IgG and CTLA4 would improve immunogenicity (Sistere-Oro et al., 2019b). Again, the epitope-based DNA vaccine led to induction of HI antibodies, although this was only measured post-challenge. The epitope-based vaccine also showed decreased levels of viral shedding post-infection after heterosubtypic H3N2 influenza challenge (Sistere-Oro et al., 2019b). This represents a promising step towards showing the potential induction of VAERD after DNA vaccination, however VAERD is typically observed through non-neutralizing antibodies developed against vaccination after H1 influenza challenge (Mancera Gracia et al., 2020). Since the challenge used an H3 influenza strain, it is inconclusive whether this vaccine design would cause VAERD after a heterologous H1 challenge.

As a vaccine delivery platform for swine, DNA vaccines show promise in inducing robust immune responses while not possessing some of the safety risks associated with other platforms such as LAIV. However, the platform has received relatively less attention as an alternative to whole-inactivated vaccines compared to the other alternative platforms. Therefore, it is likely that further understanding of the potential use for DNA vaccines against swine influenza viruses will add to the arsenal of protection against IAV-S.

### 4.3 Viral vectored vaccines

Viral vectored vaccines have recently gained traction as an attractive platform to protect against IAV-S. Viral vector vaccines can be engineered to have enhanced safety profiles and prevent viral recombination through restricted replication. Deleted regions can then be substituted with the desired vaccine immunogen. The inherent immunogenicity of the vector proteins can induce strong immune responses to the desired immunogen during transient transgene expression. Additional advantages of viral vectored vaccines include rapid production, scalability for large-scale dose manufacturing, and typically do not require eggs for propagation. These traits make viral vectors an attractive platform for producing livestock vaccines. Several viral vectors, such as Pichinde virus (PICV), Orf virus (ORFV), and Adenovirus (Ad) are currently in experimental development and are reviewed in this section and summarized in **Figure 3**.

#### 4.3.1 Pichinde virus

Pichinde virus (PICV) is a bisegmented and enveloped RNA virus within the family *Arenaviridae*. PICV was first isolated from rice rats in Colombia (Mclay et al., 2014) but can infect a wide range of cell types from humans, mice, monkeys, birds, and pigs (Dhanwani et al., 2015, 2017; Kumar et al., 2021; Kumari et al., 2022). Initial studies in mice demonstrated that PICV encoding the HA and NP proteins of A/Puerto Rico/8/34 protected mice against lethal IAV infection after two immunizations. The observed protection elicited from PICV-vectored vaccination was mediated by high levels of HA-specific neutralizing antibody responses and NP-specific CD8+ T cell responses (Dhanwani et al., 2015). Notably, antibody assessment after two or more immunizations indicated that, despite boosting with the same viral vector, antibody responses against the delivered transgene were still able to be boosted. This suggests that PICV may not induce vector-specific neutralizing antibody responses and has potential as an efficient viral vector for homologous prime-boost vaccine regimens. Recently, Kumari et al. has demonstrated that PICV can also be used as a viral vector to protect pigs against IAV-S. Two doses of recombinant PICV encoding the prototype Texas/98 HA gene (rPICV-H3) induced robust neutralizing antibody titers that were as good as immunization with recombinant H3 TX/98 protein. After homologous challenge, rPICV-H3 immunized pigs showed significantly reduced viral shedding from nasal secretions, no infectious virus recovered from the lungs, and minimal macroscopic or microscopic lung lesions compared to sham vaccinated animals. Further, pigs vaccinated with recombinant PICV encoding GFP (rPICV-GFP) did not horizontally transmit infectious rPICV to naive pigs (Kumari et al., 2022). This indicates that PICV may be a safe platform for immunization against swine pathogens with limited risk of vector circulation. While the induction of cell-mediated immune responses, induction of VAERD after heterologous challenge, breadth of protection against antigenically distinct IAV-S, or robustness against interfering MDAs were not analyzed in this study, these initial results in pigs indicate that PICV can serve as a potential viral vector against important swine pathogens and warrants further investigation.

#### 4.3.2 Orf virus

Orf virus (ORFV) is a large double-stranded DNA parapox virus belonging to the family *Poxviridae* and has been extensively evaluated as a vectored-vaccine candidate against pseudorabies (PRV) (Dory et al., 2006), porcine epidemic diarrhea virus (PEDV) (Hain et al., 2016; Joshi et al., 2018), and classical swine fever virus (CSFV) (Voigt et al., 2007). ORFV has a restricted host range, can induce both humoral and cell-mediated immune responses (Hain et al., 2016), does not elicit vector-neutralizing antibody responses that inhibit repeated immunizations (Haig and Mcinnes, 2002), and encodes several non-essential genes that can be completely or partially removed to accommodate desired vaccine immunogens. Building on previous success against several swine pathogens, researchers have recently investigated the utility of ORFV encoding a wildtype HA alone (ORFV-HA) or in combination with a wildtype NP (ORFV-HA-NP) to provide protection against a variety of H1 IAV-S isolates (Joshi et al., 2021). While robust neutralizing or total IgG antibody responses were not observed after a single immunization, boost immunization with ORFV-HA or ORFV-HA-NP elicited high neutralizing antibody titers against the homologous IAV-S. Analysis of IgG1 and IgG2 isotype antibody induction demonstrated that, while ORFV-HA induced a predominantly Th2 biased response, ORFV-HA-NP was biased towards a Th1 response, suggesting a possibility of higher cell-mediated immunity after ORFV-HA-NP immunization. Indeed, further investigation of virus-specific T cell responses revealed that immunization with ORFV-HA-NP induced higher levels of total T cells secreting IFN-γ coupled with increased proliferation of CD3^+^CD4^-^CD8^-^ and CD3^+^CD4^+^CD8^+^ T cell populations. Intranasal challenge with a homologous IAV-S showed that pigs immunized with ORFV-HA-NP had significantly reduced viral RNA in nasal swabs at 1- and 3-days post challenge compared to ORFV-HA, suggesting that the higher cell-mediated responses are playing a role in the rapid clearance of virally infected cells (Grant et al., 2013). To assess this vaccine’s potential as a candidate universal IAV-S vaccine, neutralizing antibody responses against genetically and antigenically distinct H1 IAV-S clades were analyzed. Serum from ORFV-HA immunized pigs was shown to neutralize only two out of ten divergent IAV-S analyzed, while serum from ORFV-HA-NP immunized pigs neutralized six out of the ten analyzed strains (Joshi et al., 2021). These data indicate that, while both ORFV-HA and ORFV-HA-NP can induce robustly neutralizing antibody responses against heterologous IAV-S, the inclusion of the NP gene can enhance the breadth of neutralizing antibody responses against divergent IAV-S. While this vector platform is still in the beginning stages of vaccine development against IAV-S, this study highlights the potential of ORFV as a promising vector for important swine pathogens. Additional research into protection against heterologous challenge would reveal the ability to use ORFV as a platform against IAV-S and ability to circumvent VAERD.

#### 4.3.3 Adenovirus

Belonging to the family *Adenoviridae*, adenovirus (AdV) vectors have long been appreciated for their utility against various human and animal pathogens. Adenoviruses have linear double-stranded DNA genomes and deletion of the E1A early gene can render AdV replication-defective to enhance the safety profile of this vaccine modality. A desired vaccine immunogen can then be inserted into the E1A region to develop AdV vectors against various swine pathogens (Sakurai et al., 2022). Indeed, previous research has utilized AdV to successfully immunize swine against pseudorabies virus (Eloit et al., 1990; Monteil et al., 2000), foot-and-mouth disease (Mayr et al., 1999), and PRRSV (Hernandez et al., 2017; Xie et al., 2019). AdV vectors are inexpensive to produce in mass quantities and can induce both humoral and cell-mediated immune responses after vaccination. Wesley, et al. demonstrated that intramuscular vaccination with an adenovirus type 5 expressing an H3N2 HA alone (AdV5-HA) or the HA and NP protein together (AdV5-HA+NP) induced robust HI antibody responses against the homologous virus. Notably, pigs vaccinated with AdV5-HA or AdV5-HA+NP were completed protected against viral shedding and had significant reduction of lung lesions after challenge, while pigs vaccinated with NP alone (AdV5-NP) were not protected from challenge (Wesley et al., 2004). These data indicate that the antibodies elicited after vaccination with HA alone or with another immunogen can induce strong protective immunity when delivered by an adenoviral vector. A separate study also characterized that, unlike traditional inactivated virus vaccines, AdV5 expressing IAV-S immunogens are not susceptible to neutralization by interfering MDAs (Wesley and Lager, 2006). This is a significant finding because MDAs are a common source of vaccine failure in young suckling or nursey-age pigs. While intramuscular immunization with AdV typically induces systemic responses, intranasal vaccination with AdV5 expressing an H1N1pdm09 HA (AdV5-HA) in swine demonstrates that this route of immunization can induce complete protection against homologous challenge and partial protection against heterologous challenge, while also preventing the induction of VAERD (Braucher et al., 2012). Protection against homologous challenge was likely mediated by a combination of mucosal IgA responses and antigen-specific cell-mediated immunity, while the protection against the heterologous challenge virus was likely due to the development of cross-reactive IFN-γ producing T cell responses targeting conserved epitopes in the HA protein (Gutierrez et al., 2016). Further, Petro-Turnquist et al. has recently described the kinetics and duration of humoral and cell-mediated immunity after vaccination with an adenoviral-vectored H3N2 IAV-S vaccine compared to a commonly used WIV vaccine. While vaccination with WIV did not induce robust cross-reactive antibody responses until after the second dose of vaccination, a single immunization with the AdV-vectored vaccine rapidly induced robustly cross-reactive antibodies against representative strains from H3N2 Cluster IV(A-F) and the 2010.1 human-like clade. Notably, these cross-reactive antibody responses were boosted after a second immunization three weeks after the initial dose, and these responses remained durable for up to 6 months after the initial vaccination. Further, WIV induced modest IFN-γ T cell responses that quickly diminished while the IFN-γ T cell responses elicited after AdV vaccination remained detectable for the full duration of the study. Heterologous challenge with a divergent IAV-S isolate 6 months after the initial immunization demonstrated that pigs vaccinated with AdV had a significant reduction in infectious virus in the lungs, microscopic lung and trachea lesions, and a reduced duration of viral shedding compared to WIV and unvaccinated controls (Petro-Turnquist et al., 2023). The easy and rapid development of AdV vectored vaccines, rapid and balanced induction of antibody and T cell responses, resistance to MDA interference, and ability to elicit long-lasting immunity in pigs indicates that this may be a useful platform for a universal IAV-S vaccine.

### 4.4 Computationally designed immunogen vaccines

Recent advances in immunoinformatics have contributed to rationally designed, computationally derived IAV-S vaccines. While a majority of current and preclinical IAV-S vaccines encode sequences derived from wildtype field isolates, newer developments are utilizing computational modeling to produce single or multivalent vaccine immunogens against IAV-S. This strategy aims to improve current vaccination methods by immunizing with epitopes that are predicted to elicit enhanced cross-reactive antibody or T cell responses. Different strategies that have been taken to achieve this goal will be reviewed below and are summarized in **Figure 3**.

#### 4.4.1 Consensus design

Consensus immunogen designs against human IAV have been reviewed previously (Bullard and Weaver, 2021), and this tactic has also been adapted into IAV-S vaccine development. Consensus vaccines are developed by computing the most common amino acid at each position along the target protein. The approach aims to decrease the genetic distance between the vaccine and circulating field strains by creating a synthetic immunogen that is representative of a diverse population of sequences. Sun H., et al. recently described the utility of a consensus vaccine immunogen against H3 IAV-S HA protein (H3-CON.1) (Sun et al., 2019). Analysis of several genetically diverse IAV-S H3 strains demonstrated that the consensus immunogen induced significantly higher cross-reactive antibody and T cell responses compared to a wildtype HA (H3-TX98). Importantly, after challenge with a heterologous IAV-S isolate, vaccination with H3-CON.1 significantly reduced the severity and duration of viral shedding in the nasal secretions compared to the H3-TX98 wildtype HA. These results indicate that a monovalent consensus vaccine design can improve cross-reactive responses against divergent H3 IAV-S compared to a wildtype sequence alone. Similarly, a consensus vaccine targeting H1 IAV-S has recently been described (Do Nascimento et al., 2023). The consensus H1 IAV-S immunogen was developed using IAV-S sequences isolated between 2014-2017 and delivered by an ORFV viral vector (ORFV-conH1). Compared to unimmunized controls, ORFV-conH1 induced significantly higher IgG antibody levels against representative IAV-S strains from the α, β, γ, δ1, γ-2-β-like, and new pandemic (npdm) clades. While promising, it is currently unknown if the developed antibodies had the capacity to efficiently neutralize divergent H1 IAV-S, as this was not reported in this study. Nonetheless, heterologous challenge with a γ clade IAV-S revealed that pigs immunized with ORFV-conH1 had significantly higher levels of IFN-γ secreting CD8^+^ T cells in the bronchioalveolar lavage (BAL) following challenge. These T cell responses likely played a role in clearing viral infected cells, as ORFV-conH1 immunized pigs showed a significant reduction of infection virus in nasal secretions at 2- and 6-days post challenge and lower levels of viral RNA in the BAL and lungs at the conclusion of the challenge. In contrast, immunization with ORFV-conH1 and challenge with a npdm clade virus induced a significantly higher frequency of T cells with a helper/memory phenotype and circulating IFN-γ CD8^+^ T cells. This induction of cell-mediated immune responses likely in the absence of neutralizing antibody responses again translated to a significant reduction of infectious virus in nasal secretions and 2- and 6-days post challenge coupled with lower viral RNA in the BAL (Do Nascimento et al., 2023). Currently, reasons driving the differential kinetics of T cell development in these anatomical compartments are unknown, but analysis of the T cell responses prior to challenge may help uncover the reason behind these differences. Further, given that the ORFV-conH1 was not compared to a wildtype IAV-S H1 sequence, it is currently unclear how the consensus design may enhance cross-reactive responses against H1 IAV-S and more research into this comparison is necessary to determine the breadth of protection against H1 IAV-S.

#### 4.4.2 PigMatrix design

Recent studies have focused on harnessing adaptive cell-mediated immune responses to combat IAV-S in pigs. In humans, T cell activation is mediated through engagement of the T cell receptor (TCR) with peptides bound to the human leukocyte antigen (HLA) expressed on the surface of antigen presenting cells. The similar structural and pocket-binding characteristics of HLA and swine leukocyte antigen (SLA) molecules provides an opportunity to leverage known human IAV epitopes to predict potentially immunogenic IAV-S epitopes. PigMatrix is a novel computational model that utilizes datasets of SLA binding peptides, SLA structures, and known HLA binding preferences to predict important class I and class II SLA epitopes for robust T cell activation in pigs. Ideally, pigs can be surveyed for common SLA haplotypes and used for epitope prediction. The resulting predicted SLA class I and SLA class II epitope sequences can then be synthesized as peptides or delivered as a DNA vaccine to enhance T cell activation after immunization and induce more broadly reactive responses against IAV-S (Gutierrez et al., 2015). A recent study evaluated the utility of PigMatrix to predict potentially immunogenic class I and class II IAV-S epitopes using SLA haplotypes common in U.S. pig populations. Pigs were immunized with DNA vectors encoding 48 class I and class II epitopes of both internal and external IAV-S proteins, 26 class I and class II epitopes from internal IAV-S proteins, 8 class II peptides from external IAV-S proteins, or 14 class I peptides from internal IAV-S proteins. Though, retrospective analysis of the cohort of pigs used in the study revealed mismatched SLA allelic frequency from the SLA alleles used to predict immunogenic epitopes, several promiscuous IAV-S epitopes were identified in the HA, NA, M, PA, and PB genes. Additionally, they paradoxically identified that robust IFN-γ T cell responses to class I peptides were restricted to external peptides, and IFN-γ T cell responses to class II peptides were seen against internal peptides (Gutierrez et al., 2016). This is in stark contrast to humans, where class I epitopes are typically found in internal proteins and class II epitopes are commonly restricted to external IAV proteins. Based on these results, additional *in vivo* validation is necessary to narrow the predictive efficacy of this computational model. Notably, given that this tactic of vaccine development aims to enhance cross-reactive T cell responses, immunization with the short peptide sequences often precludes important B cell epitopes and dampens or completely abrogates antibody development (Gutierrez et al., 2016). However, priming with the predicted SLA class I and class II T cell epitopes and boosting with a commercial WIV vaccine may be able to induce a balanced antibody and T cell response for optimal protection against homosubtypic IAV-S challenge (Hewitt et al., 2019). While the PigMatrix platform is still under intense investigation, these promising experimental results can be a useful in developing computational tools for rationally designed vaccines against various swine pathogens.

#### 4.4.3 Epigraph design

Epigraph is a recently described method of synthetic immunogen design that maximizes potential T cell epitopes incorporated into a vaccine design. This platform uses a graph-based epitope optimization approach to create a cocktail of vaccine immunogens with enhanced potential epitope coverage of a highly diverse population of sequences. The Epigraph method was initially created to develop broadly reactive vaccine immunogens against HIV (Theiler et al., 2016; Theiler and Korber, 2018) and our group has recently utilized the Epigraph design to prevent H3 IAV (Bullard et al., 2022) and IAV-S infections (Bullard et al., 2021). Bullard, B. et al. demonstrated that Epigraph immunogens targeting swine H3 HA can induce high levels of cross-reactive antibody and T cell responses that are significantly better than a wildtype HA sequence and a commercial vaccine. Importantly, to analyze the potential of the Epigraph platform as a universal IAV-S vaccine, the Epigraph was analyzed against 20 different H3N2 strains spanning multiple decades and clades of IAV-S. When pigs were immunized with a single dose of Epigraph, they mounted protective antibody responses against 13 out of the 20 representative strains, while the wildtype HA only induced protective antibodies against the homologous strain and the commercial comparator vaccine failed to induce protective antibody responses after a single dose. These antibody responses were coupled with robust cross-reactive T cell responses against four heterologous IAV-S strains. Importantly, given that swine are also susceptible to human IAV, the serum from immunized pigs were analyzed for cross-reactive antibody responses against 7 divergent human IAV strains. A single immunization with Epigraph elicited protective antibody responses against 3 out of 7 of the human IAV strains, while immunization with a wildtype HA provided protective antibodies against 1 out of the 7 analyzed human IAV strains and commercial comparator vaccine did not elicit protective antibodies against any of the human IAV strains after a single immunization. This study further analyzed protection against challenge with 3 divergent IAV-S strains and 1 human IAV strain in mice. Epigraph immunized mice had the lowest weight loss and a significant reduction of infectious virus in the lungs compared to immunization with a wildtype HA or the commercial comparator vaccine. While these experimental results in mice are encouraging for continued analysis of the Epigraph vaccine, additional research detailing protection against divergent IAV-S challenges in pigs are necessary to further assess this platform as a candidate universal IAV-S vaccine.

### 4.5 Nanovaccines

Nanovaccines have recently emerged as a powerful tool to selectively deliver antigens to mucosal surfaces and facilitate rapid and robust immunity against various infectious diseases (Sadr et al., 2023). Nanovaccines aim to protect the encapsulated vaccine antigens against degradation in physiological environments and utilize slow release of the cargo for prolonged immune activation. Further, this vaccine platform can be conjugated with ligands to improve uptake of the vaccine antigen by professional antigen presenting cells (Sadr et al., 2023). Delivery to the site of infection, the respiratory mucosal interface, can lead to improved adaptive immune activation and robust development of secretory IgA (sIgA) and local T cell responses against the delivered antigen to improve clinical outcomes (Chadwick et al., 2010). Here, we review polyanhydride, poly(lactic-*co*-glycolic acid) (PLGA), and chitosan nanovaccines and summarize these advances in **Figure 3**.

#### 4.5.1 Polyanhydride nanovaccines

Polyanhydride is a biocompatible and biodegradable polymer that can be used to encapsulate antigens while retaining the biological and structural properties of the vaccine antigens. Polyanhydride nanovaccines have previously shown to elicit protective antibody responses against H5N1 IAV (Ross et al., 2015) and *Yesinia pestis* (Ulery et al., 2011) in mice and against bovine respiratory syncytial virus (BRSV) in the neonatal calf model (Mcgill et al., 2018). This strategy has recently been adapted to combat H1 IAV-S in pigs to elicit cross-reactive mucosal immunity after intranasal immunization. Dhakal et al. investigated the protective efficacy of a polyanhydride nanovaccine encapsulating a killed H1N2 IAV-S (KAg nanovaccine) compared to unencapsulated killed H1N2 IAV-S (KAg). The KAg nanovaccine promisingly induced robust antigen-specific lymphocyte proliferation that led to trends of lower clinical disease after heterologous H1N1 challenge, and a significant reduction in viral antigen in lungs compared to unvaccinated controls. The KAg nanovaccine, however, did not induce high levels of mucosal IgA in the respiratory tract or systemic HI, IgG, or virus neutralizing antibodies (Dhakal et al., 2017a). This lack of antibody response is likely due to the polyanhydride encapsulation hiding important B cell epitopes on the outside of the killed virus. To improve upon this platform, additional studies investigated a co-delivery of the KAg nanovaccine encapsulating an H1N2 isolate with a CpG-ODN TLR-9 agonist (Dhakal et al., 2019). Addition of the CpG agonist into the nanovaccine formulation improved IgA responses in nasal swabs compared to the KAg nanovaccine without CpG and the KAg and also led to higher levels of proliferating lymphocyte responses compared to KAg. Importantly, the KAg nanovaccine with CpG also induced IgA responses in nasal swabs against a heterosubtypic H3N2 isolate, indicating robust cross-reactive immune induction. Heterologous challenge with an H1N1 showed that the KAg nanovaccine with CpG had a trend of lower clinical disease and virus in nasal swabs compared to unvaccinated controls. This protection was likely mediated through a balanced induction of IgA in the respiratory tract and robust cell-mediated immunity, as the KAg nanovaccine with CpG vaccinates demonstrated higher IFN-γ producing cells collected from the tracheobronchial lymph node (TBLN) against the homologous H1N2 virus, heterologous H1N1 virus, and heterosubtypic H3N2 following challenge (Dhakal et al., 2019). These promising initial studies indicate that polyanhydride encapsulation with additional adjuvants can be effective in preventing infection of heterologous IAV-S in pigs and warrants further investigation using heterosubtypic challenge models.

#### 4.5.2 Poly(lactic-*co*-glycolic acid) nanovaccines

Poly(lactic-*co*-glycolic acid (PLGA) polymers are a class of FDA-approved polymers that have been widely used to develop a variety of vaccines and therapeutics (Lu et al., 2009; Binjawadagi et al., 2014). PLGA can be used to encapsulate whole virus antigens, drug products, and peptides then administered intranasally to induce local mucosal immunity. Indeed, a recent study has analyzed the protective efficacy of encapsulating conserved H1N1 IAV peptides from the HA, NP, PA, and M2e to induce broadly cross-reactive responses in swine (Hiremath et al., 2016). The PLGA-encapsulated peptide cocktail induced significant levels of IFN-γ secreting CD4^+^ helper memory and cytotoxic T cells against the NP, PA, and HA peptides. As expected with peptide antigens that often preclude important B cell epitopes, pigs vaccinated with the PLGA-encapsulated peptide vaccine had low IgG and IgA antibody titers in the serum at and mucosal sites. Nonetheless, pigs vaccinated with the PLGA-encapsulated peptide vaccine were protected from clinical disease and had no detectable infectious virus in the lungs following heterologous H1N1 challenge (Hiremath et al., 2016), suggesting that the protection may have been mediated by T cell responses. In a subsequent study by Dhakal et al., a similar strategy of PLGA encapsulation was used to deliver killed H1N2 IAV-S (PLGA-KAg) intranasally and compared to killed H1N2 (KAg) alone (Dhakal et al., 2017b). The PLGA-KAg showed the ability to increase CD80/86 expression on porcine dendritic cells and macrophages *in vitro* compared to KAg alone, indicating improved maturation of these major antigen presenting cells. Indeed, this improved maturation led to significantly higher rates of lymphocyte proliferation after stimulation with either H1N2 or H1N1 in the PLGA-KAg immunized pigs compared to immunization with the KAg alone. Upon heterologous challenge with an H1N1 IAV-S isolate, pigs immunized with PLGA-KAg showed reduced duration of clinical disease, a trend of lower macroscopic and microscopic lung lesions, and lower presence of infectious virus in the lungs compared to KAg. This improved protection was likely due to higher IFN-γ secreting recall CTL responses and moderate levels of IgA in the BAL following challenge (Dhakal et al., 2017b). While this platform has promisingly shown protection against heterologous challenge and does not indicate induction of VAERD, more research is required to characterize if this platform will still be effective in the presence of MDA.

#### 4.5.3 Chitosan nanovaccines

Chitosan is a class of biodegradable and biocompatible cationic polysaccharide that exhibits mucoadhesive properties as the positively charged amino acid side chains interact with negatively charged sialic acid receptors in the respiratory tract (Tm et al., 2018). This results in directed delivery of vaccine antigens, minimal degradation of the encapsulated antigen, and longer duration of immune activation after administration. Recent studies have characterized the efficacy of intranasally delivering chitosan encapsulated killed H1N2 IAV-S (CNPs-KAg) compared to killed H1N2 IAV-S (KAg) alone (Dhakal et al., 2018). The CNPs-KAg construct was shown to activate porcine dendritic cells *in vitro* and elicited significantly higher levels of IgA in nasal swabs against the homologous H1N2, heterologous H1N1, and a heterosubtypic H3N2 isolate compared to KAg. Further, the CNPs-KAg immunized pigs mounted significantly higher IgA antibody responses in the nasal swabs, BAL, and lung lysate and significantly higher IgG in the serum compared to unvaccinated pigs. While the CNPs-KAg demonstrated higher levels of IFN-γ secreting cellular responses, this did not necessarily lead to improved clinical outcomes, as there were similar trends of fever, macroscopic lung lesions, and proinflammatory cytokine IL-6 among all vaccine groups following heterologous H1N1 challenge (Dhakal et al., 2018). To improve upon this vaccine, an additional study investigated the utility of including the TLR3 agonist, poly(I:C), to enhance innate and adaptive immune responses and compared this strategy to a commercial comparator vaccine, FluSure XP (Renu et al., 2020). While inclusion of poly(I:C) into the chitosan nanovaccine formulation may potentially improve antibody and cell-mediated immunity after immunization, it is challenging to definitively conclude this because the chitosan nanovaccine encapsulating the inactivated H1N2 with poly(I:C) was not directly compared to the chitosan nanovaccine without poly(I:C). Nonetheless, including poly(I:C) into the chitosan nanovaccine appeared to improve induction of circulating HI antibody responses when compared across studies (Dhakal et al., 2018), and resulted in similar clinical outcomes achieved by the commercial FluSure XP vaccine (Renu et al., 2020). Finally, to further improve this platform Renu et al. has investigated mannose conjugation to chitosan nanoparticles encapsulating killed H1N2 IAV-S (mCS-KAg) in the presence of MDAs (Renu et al., 2021). Pigs immunized with mCS-KAg had robust IgA induction in nasal swabs against H1N2, heterologous H1N1, and against H3N2. This conjugated vaccine also induced increased recall lymphocyte proliferation and IL-4, IL_10 and IFN-γ gene expression compared to the unconjugated chitosan nanoparticle vaccine (CS-KAg) and commercial vaccine. Consequently, after heterologous H1N1 infection both the mCS-KAg and CS-KAg cleared infectious virus from the upper and lower respiratory tract more readily than the pigs immunized with FluSure XP and had reduced macroscopic lung lesions (Renu et al., 2021). Overall, chitosan nanoparticle vaccines offer the potential to induce robust cross-reactive mucosal IgA and systemic IgG responses, coupled with balanced cell-mediated immunity to provide protection against heterologous infection in the presence of MDA.

## 5 Conclusions

IAV-S is one of the most important swine pathogens due to the economic burden imposed on the pork industry and its zoonotic potential. Vaccination remains the most effective method of controlling or preventing IAV-S in swine. Despite continued efforts in the development of a broadly protective vaccine against IAV-S, there are extensive challenges to achieving this goal. Though, only three subtypes of IAV-S circulate in swine populations, each subtype demonstrates extensive genetic and antigenic diversity. Vaccine-elicited antibody and T cell responses are relatively understudied in pigs compared to human and mice, and further research into this area can contribute to our understanding of important immunodominant B and T cell epitopes. Further, interference of MDA and the induction of VAERD after vaccination often inhibit protection afforded by commercial vaccination strategies. Indeed, current vaccines against IAV-S struggle to address the constantly evolving nature of IAV-S, fail to provide broad protection and require custom-made vaccines to better represent strains circulating in a given herd, but are often hampered by slow production times and immune-mediated interference resulting in suboptimal protection.

To improve upon these shortcomings, researchers are investigating novel vaccine platforms, vector design, utilizing computationally designed vaccine immunogens, and pursuing new-age nanovaccine technologies to increase cross-reactive responses against IAV-S. These experimental vaccine strategies aim to induce better heterologous and even heterosubtypic protection against H1 and H3 IAV-S compared to existing licensed vaccines. While this is the general goal of experimental vaccines, a majority of studies often fail to directly compare the experimental vaccine with a licensed commercial comparator vaccine. Future studies that include this side-by-side comparison may improve our understanding of vaccination methods that elicit broadly cross-reactive responses and expedite the process of licensing novel vaccine strategies. There are several platforms, such as live attenuated influenza virus vaccines, Orf virus-vectored, adenovirus-vectored, computationally designed Epigraph and consensus vaccine strategies, and mannose conjugated chitosan nanovaccines, that induce a balanced antibody and T cell responses after immunization. Of these platforms, adenoviral vectored and mannose conjugated chitosan nanovaccines have been described to be resistant to interference of MDA. However, it is possible that the other experimental vaccine strategies described here are also resistant to MDA interference, but simply have not been evaluated yet, providing a multitude of knowledge gaps yet to be explored. Finally, VAERD induction is an important and previously unforeseen challenge in developing a universal IAV-S vaccine. Initial testing of these experimental vaccines often investigate protection against homologous strains included in the vaccine so additional studies using heterologous challenges will further undercover the induction of VAERD in future studies. While these novel vaccine platforms are still in the beginning stages of experimental development, preliminary research indicates that we are getting closer to achieving the goal of a universal IAV-S vaccine.

## Author contributions

EP-T: Conceptualization, Visualization, Writing – original draft, Writing – review & editing. MP: Writing – original draft, Writing – review & editing. EW: Funding acquisition, Supervision, Writing – original draft, Writing – review & editing.
